# Super-long-day treatment bypasses vernalization and induces flowering directly in *Brassica rapa*
and *Raphanus sativus*

**DOI:** 10.1270/jsbbs.25083

**Published:** 2026-06-20

**Authors:** Shinsuke Yasuda, Takayuki Ogami, Kazuo Ide, Hiroshi Tanaka

**Affiliations:** 1 Plant Breeding and Experiment Station, Takii and Company, Limited, Konan, Shiga 520-3231, Japan

**Keywords:** *Brassica rapa*, *Raphanus sativus*, flowering, long day, vernalization

## Abstract

Flowering promotion is an essential technique in plant breeding. In plants of the Brassicaceae family, flowering generally requires a prolonged period of low temperature, known as vernalization, which often represents a rate-limiting step in breeding programs. In this study, we identified a phenomenon termed Long-day Induced Vernalization Exclusion (LIVE), in which flowering is triggered under non-conventional long-duration light conditions without the need for vernalization. In *Brassica rapa* and *Raphanus sativus*, this phenomenon was observed across many lines under super-long-day conditions, and its induction became increasingly pronounced as the day length extended. LIVE was also induced under a repeating 11-hour light/1-hour dark cycle, suggesting that uninterrupted long day length is not strictly required for LIVE induction. This study reveals that low-temperature treatment can be bypassed through these long-duration light conditions across multiple lines, offering important insights for accelerating flowering in practical breeding.

## Introduction

In the Brassicaceae, flowering requires a period of low-temperature treatment known as vernalization. The flowering pathway in this family has been extensively studied using the model plant *Arabidopsis thaliana*. Flowering is intricately regulated by multiple genetic pathways, including the vernalization, photoperiod, autonomous, and gibberellin pathways (Leijten *et al.* 2018).

Among these, the vernalization pathway is primarily governed by two key genes, *FLC* (*FLOWERING LOCUS C*) and *FRI* (*FRIGIDA*), which play major roles in determining flowering time in *A. thaliana* (Shindo *et al.* 2005). *FLC* encodes a MADS-box transcription factor that represses downstream flowering genes, including the florigen gene *FT* (*FLOWERING LOCUS T*), thereby inhibiting flowering (Helliwell *et al.* 2006, Searle *et al.* 2006). *FRI* promotes *FLC* transcription and thus delays flowering (Choi *et al.* 2011). After prolonged low-temperature exposure, *FLC* is epigenetically silenced, leading to flowering induction (Bastow *et al.* 2004). In *Brassica oleracea*, *Brassica rapa*, and *Raphanus sativus*, allelic variation in these genes critically influences flowering time (Hou *et al.* 2025, Kitamoto *et al.* 2014, Wang *et al.* 2018).

In the photoperiod pathway, *CONSTANS* (*CO*) plays a central role. CO protein acts as a transcriptional activator of *FT* (Samach *et al.* 2000), and it’s stability is higher under long-day than under short-day conditions (Valverde *et al.* 2004).

In Brassicaceae vegetables, premature bolting reduces market value, so breeding for late-bolting cultivars that are tolerant to low temperatures has been a key objective. However, these late-bolting lines usually require longer cold exposure, extending the breeding cycle. Promotion of flowering is therefore essential for accelerating the development of such cultivars. Several attempts to bypass vernalization have been reported, though few have been successful. In *Brassica napus*, continuous gibberellin treatment—a phytohormone associated with flowering—induces floral bud formation without vernalization (Lang 1957). Similarly, in the vernalization-dependent species *Thlaspi arvense*, flowering was triggered by applying 5-azacytidine, a DNA methylation inhibitor (Burn *et al.* 1993). Moreover, grafting a *B. oleracea* scion onto a flowering *R. sativus* rootstock also induced flowering without vernalization (Motoki *et al.* 2019). However, these methods have not yet been widely applied in breeding processes, partly due to concerns regarding labor requirements and chemical toxicity.

In 2018, the Lee group at the University of Queensland reported a method known as speed breeding, in which flowering is promoted under super-long-day conditions such as a 22-hour photoperiod (Watson *et al.* 2018). This approach has enabled generation acceleration in many crop species. However, the study primarily focused on lines that do not require vernalization. Consequently, its effectiveness on vernalization-requiring genotypes remained unclear. The vernalization-requiring *B. rapa* cultivar ‘Leafy green parental line No. 2 (Tsukena No. 2)’ has flowered under a 16-hour photoperiod without vernalization, although this trait had been considered specific to this cultivar (Yui *et al.* 2003). In this study, we report the discovery that *B. rapa* and *R. sativus*, both of which normally require vernalization to flower, can instead flower without cold treatment when grown under a 22-hour photoperiod, not only in ‘Tsukena No. 2’ but also in other cultivars.

## Materials and Methods

### Growth conditions and evaluation of flowering date

Seeds of 50 commercial cultivars of *B. rapa* and 43 of *R. sativus* (Supplemental Tables 1, 2) were sown in square pots (5 cm per side) filled with a commercial seedling soil mix (Seru-Baido TM-1; Takii Seed, Kyoto, Japan). The medium was supplemented with chemical fertilizers to adjust the nutrient composition to 150 mg/L N, 110 mg/L P_2_O_5_, and 260 mg/L K_2_O and grown at a constant temperature of 22°C under a 22-hour photoperiod (Zeitgeber time [ZT] 0 is defined as the time of lights-on; lights were on from ZT0 to ZT22 and off from ZT22 to ZT24). From two weeks after sowing, plants were supplied with a 500-fold dilution of a liquid fertilizer (Kumiai Liquid Fertilizer #2, Katakura & Co-op Agri Corporation) via subirrigation once per week. Plants were grown in a growth chamber under LED lighting with a mean photosynthetic photon flux density (PPFD) of 269 ± 58 μmol m^–2^ s^–1^ (mean ± standard deviation [SD]). The illuminance was 17358 ± 3715 lux. Spectral photon flux densities were 58 ± 13 μmol m^–2^ s^–1^ (blue light; PFD-B, 400–500 nm), 110 ± 23 μmol m^–2^ s^–1^ (green light; PFD-G, 500–600 nm), 102 ± 21 μmol m^–2^ s^–1^ (red light; PFD-R, 600–700 nm), and 68 ± 24 μmol m^–2^ s^–1^ (far-red [FR] light; PFD-FR, 700–780 nm). The R/FR ratio was calculated as the sum of PFD at 600–700 nm divided by that at 700–780 nm. A light analyzer (LA-105, NK System) was used to measure light intensity and spectral distribution at the height of the plant growth trays at five positions (center and four corners) within the growth chamber. The experiment was terminated 83 days after sowing. For six lines each of *B. rapa* and *R. sativus*, seeds were similarly sown in 5-cm square pots and cultivated at 22°C under photoperiods of 16 (lights were on from ZT0 to ZT16 and off from ZT16 to ZT24), 17 (lights were on from ZT0 to ZT17 and off from ZT17 to ZT24), 18 (lights were on from ZT0 to ZT18 and off from ZT18 to ZT24), 20 (lights were on from ZT0 to ZT20 and off from ZT20 to ZT24), or 22 hours (lights were on from ZT0 to ZT22 and off from ZT22 to ZT24) of light, or under 11 hours of light followed by 1 hour of darkness (lights were on from ZT0 to ZT11, off from ZT11 to ZT12, on from ZT12 to ZT23 and off from ZT23 to ZT24). The experiment was terminated on the 107^th^ day after sowing. Days to flowering (DtF) was defined as the number of days from sowing to the opening of the first flower.

## Results

### Discovery of vernalization-independent flowering under super-long-day conditions

To examine the effect of speed breeding on vernalization-dependent crops, we cultivated the *B. rapa* cultivar ‘Muso’ and the *R. sativus* cultivar ‘Taibyo-Sobutori’ at a constant temperature of 22°C under a 22-hour photoperiod. Remarkably, despite the absence of cold exposure normally required for reproductive transition in *B. rapa* and *R. sativus*, flowering occurred within 80 days after sowing under the 22-hour photoperiod (Fig. 1). In contrast, as expected, no flowering was observed under control conditions of a 16-hour photoperiod at 22°C. We termed this phenomenon as Long-day Induced Vernalization Exclusion (LIVE), wherein vernalization-dependent plants flower without cold treatment under super-long-day conditions.

To assess how widely LIVE occurs across species, we tested 50 accessions of *B. rapa* and 43 accessions of *R. sativus* under a 22-hour photoperiod. In *B. rapa*, LIVE was observed in 30 of the 50 accessions within 83 days after sowing (Table 1). The phenomenon was common in *pekinensis* (9 of 12 accessions), *chinensis* (4 of 5), and others (8 of 10) but less frequent in *ssp. perviridis* (4 of 8), *nipposinica* (2 of 6), and *rapa* (3 of 9). In *R. sativus*, however, LIVE was confirmed in over 90% of the accessions examined (Table 2). These findings indicate that LIVE is not restricted to specific cultivars such as ‘Muso’ and ‘Taibyo-Sobutori’ but is broadly present across *B. rapa* and *R. sativus* genotypes.

### Specific photoperiod lengths are required to induce LIVE

To determine the minimum photoperiod required to induce LIVE, we examined flowering induction under 20-, 18-, 17-, and 16-hour photoperiods. Six accessions each of *B. rapa* and *R. sativus* were selected to capture a wide range of variations in flowering earliness under 22-hour photoperiods. These included *B. rapa* accessions ‘Muso’ (35 DtF under 22-hour light), ‘Osaka-shirona’ (38.6 DtF), ‘Chihiri-70’ (40 DtF), ‘Kigokoro-85’ (42.4 DtF), ‘Haruwarai’ (54.2 DtF), and ‘Eikun’ (69 DtF), and *R. sativus* accessions ‘Haru-no-Kami’ (39.2 DtF), ‘Hanashirazu-Tokinashi’ (51 DtF), ‘Taibyo-Sobutori’ (52.4 DtF), ‘Aki-Komachi’ (54.8 DtF), ‘Natsu-Tsukasa’ (63.4 DtF), and ‘Sakurakaze’ (66.6 DtF). ‘Tsukena No. 2’, previously reported to flower without vernalization under a 16-hour photoperiod (Yui *et al.* 2003), was excluded from this experiment. A typical trend was observed in which longer photoperiods increased the frequency of LIVE induction (Table 3). The shortest photoperiod at which LIVE occurred was 16 hours, as seen in *B. rapa* accession ‘Osaka-shirona’.

Next, to test whether continuous light exposure or the duration of the dark phase was critical for LIVE induction, we grew plants under a repeating cycle of 11 hours of light followed by 1 hour of darkness. Intriguingly, LIVE was still induced under this 11-hour-light/1-hour-dark regime (Table 3). However, in several accessions, the flowering rate was lower than under continuous 22-hour light, indicating that uninterrupted long photoperiods may be more effective for LIVE induction. Taken together, LIVE was induced under a repeating 11-hour light/1-hour dark cycle, although flowering rates were reduced in several accessions, indicating that a 22-hour light/2-hour dark condition is not strictly required.

## Discussion

In this study, we discovered that two agriculturally important vernalization-requiring crops, *B. rapa* and *R. sativus*, can flower without vernalization under super-long-day conditions. Conventionally, artificial flowering induction in these species requires 1–2 months of cold treatment, which has long been a rate-limiting step in the breeding process. Previous reports have described flowering induction without vernalization through continuous treatment with gibberellin or the DNA methylation inhibitor 5-azacytidine, as well as by grafting onto a rootstock in which flowering had already been induced (Burn *et al.* 1993, Lang 1957, Motoki *et al.* 2019). The LIVE phenomenon reported here provides a low-effort and relatively simple alternative with broad applicability to breeding, at least in *B. rapa* and *R. sativus*.

In the Brassicaceae, flowering is normally repressed by *FLC* until plants experience prolonged low temperatures. During winter, *FLC* expression is epigenetically suppressed, and as day length increases in spring, activation of the photoperiod pathway eventually promotes flowering. We hypothesize that under super-long-day conditions, strong activation of the photoperiod pathway can override repression by the vernalization pathway, enabling flowering even without cold exposure.

A previous study reported inflorescence formation rates of 50%–75% in ‘Wakayama’ and 12.5% in ‘Osaka-Shijunichi’ under a 24-hour photoperiod without vernalization (Cheon *et al.* 2004). Conversely, we observed flowering rates of 5/5 and 4/5 in ‘Wakayama’ and ‘Osaka-Shijunichi’, respectively, under a 22-hour photoperiod. Light quality further influenced such differences, as our experiments employed LED and FR LED lighting (approximately R/FR = 1.5). Considering that R/FR ratio manipulation through FR enrichment generally promotes flowering (Song *et al.* 2018), supplemental FR illumination may have contributed to the improved LIVE induction.

A similar response under a 16-hour photoperiod was previously observed in specific *B. rapa* lines such as ‘Tsukena No. 2’, which was considered genotype-specific (Yui *et al.* 2003). In this study, we found that ‘Osaka-Shirona’, a known progenitor of ‘Tsukena No. 2’, also exhibited LIVE under a 16-hour photoperiod. These genotypes, which displayed LIVE under relatively short days, are likely to be highly sensitive to photoperiodic cues. In contrast, genotypes in which LIVE was not induced may possess photoperiod pathways that are less responsive or nonfunctional.

Moreover, LIVE was induced under a repeating 11-hour light/1-hour dark cycle, despite less efficiency than under continuous 22-hour light. This indicates that uninterrupted continuous light is not strictly required. A more critical involvement of the duration of the dark period or the total amount of light perceived within a 24-hour cycle remains unclear from the present experimental design. Future experiments in which dark-period length and total light exposure are independently controlled are warranted to address this question.

In *B. rapa*, cultivars that are commercially marketed as late-bolting lines such as ‘Kiraku-70’ and ‘Sakura-Komachi’, and in *R. sativus*, cultivars such as ‘Daishi’ and ‘Top Runner’, showed lower LIVE responsiveness or required longer durations for induction under extremely long-day conditions. This finding suggests that LIVE induction depends on the balance between the repressive strength of the vernalization pathway and the sensitivity of the photoperiod pathway. Further studies are required to identify the genetic determinants of LIVE and to clarify its distribution among other vernalization-dependent crops. Altogether, the discovery of LIVE is significant both for accelerating breeding cycles and for providing new physiological insight into the mechanisms that regulate flowering in plants.

## Author Contribution Statement

SY designed the project, performed the experiments, and wrote the manuscript. TO, KI, and HT contributed to the selection of appropriate experimental materials and the establishment of experimental conditions based on their expertise. All authors revised and approved the final manuscript.

## Supplementary Material

Supplemental Table 1

Supplemental Table 2

## Figures and Tables

**Fig. 1.**
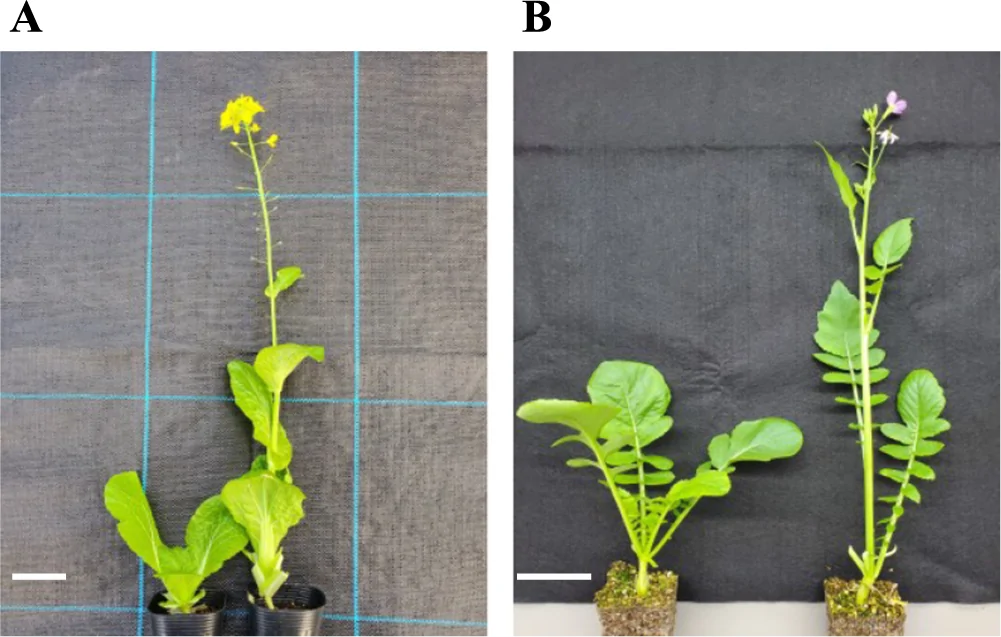
Vernalization-independent flowering under ultra-long-day conditions. (A) *Brassica rapa* (cv. ‘Muso’) and (B) *Raphanus sativus* (cv. ‘Taibyo-Sobutori’). On each panel, the plant shown on the left was grown under a 16-hour photoperiod and the plant on the right under a 22-hour photoperiod. All plants were photographed 55 days after sowing. Bar size = 5 cm.

**Table 1.** LIVE phenomenon in *Brassica rapa*

Name	ssp.	DtF (SD)	Rate
Eikun	*pekinensis*	69.0 (7.8)	5/5
Homare-no-Kiwami	*pekinensis*	69.8 (11.3)	5/5
Kiraboshi-65	*pekinensis*	82.6 (0.5)	5/5
Haruwarai	*pekinensis*	54.2 (14.1)	5/5
Kigokoro-85	*pekinensis*	42.4 (4.3)	5/5
Chihiri-70	*pekinensis*	40.0 (0)	5/5
Musou	*pekinensis*	35.0 (0)	5/5
Sakura-Komachi	*pekinensis*	N (–)	0/5
Kiraku-70	*pekinensis*	N (–)	0/5
Yuifuku	*pekinensis*	N (–)	0/5
Akimeki	*pekinensis*	53.0 (0)	5/5
Minebuki-505	*pekinensis*	41.6 (3.5)	5/5
Nanami	*perviridis*	N (–)	0/5
Natsurakuten	*perviridis*	N (–)	0/5
Nanane	*perviridis*	45.5 (10.6)	3/5
Natsu-souten	*perviridis*	48.0 (–)	1/5
Hamatsuzuki	*perviridis*	65.4 (11.5)	5/5
Sakuragi	*perviridis*	N (–)	0/5
Inamura	*perviridis*	N (–)	0/5
Tsunashima	*perviridis*	46.0 (3.0)	5/5
Entei	*chinensis*	30.6 (2.1)	5/5
Nihao-Sanka	*chinensis*	27.4 (0.5)	5/5
Nihao-114	*chinensis*	55.2 (8.7)	5/5
Natsu-Shoumi	*chinensis*	31.6 (4.0)	5/5
Taron	*chinensis*	N (–)	0/5
Kyo-Kanade	*nipposinica*	N (–)	0/5
Miyako-musume	*nipposinica*	N (–)	0/5
Kyo-Sudare	*nipposinica*	N (–)	0/5
Kyo-Dayori	*nipposinica*	N (–)	0/5
Gokuwase-suiten	*nipposinica*	56.5 (10.6)	4/4
Mibuna	*nipposinica*	39.6 (0.9)	5/5
Kyo-Senmai	*rapa*	47.5 (0.6)	4/5
Taibyo-Hikari	*rapa*	36.0 (3.1)	5/5
Swan	*rapa*	39.8 (2.0)	5/5
CR Mochibana	*rapa*	N (–)	0/5
CR Koibana	*rapa*	N (–)	0/5
CR Yukibana	*rapa*	N (–)	0/5
TKA-769	*rapa*	N (–)	0/5
Fuku-Komachi	*rapa*	N (–)	0/5
ARAMIS	*rapa*	N (–)	0/5
Tsukena No. 2	others	29.4 (2.2)	5/5
Nozawana	others	73.3 (15.0)	3/5
Tokinashi-Taisai	others	N (–)	0/5
Seppaku-Taisai	others	47.2 (12.9)	5/5
Taasai	others	40.2 (12.6)	5/5
Shinbansei-Aburana	others	N (–)	0/4
Shiroguki-Santousai	others	53.6 (15.6)	5/5
Tokyo-Bekana	others	47.8 (12.1)	4/4
Osaka-shirona	others	38.6 (12.6)	5/5
Hikoshima-Haruna	others	51.2 (18.1)	5/5

Table presents the date of first flowering within 83 days after sowing. “DtF: Days to Flower” indicates the average number of days to flowering based on the flowering individuals among the individuals used. “N” indicates that flowering did not occur. SD was calculated only for accessions in which three or more plants flowered; when two or fewer plants flowered, SD was not calculated and is indicated by “–” in the table. “Rate” represents number of flowering plants out of the individuals used.

**Table 2.** LIVE phenomenon in *Raphanus sativus*

Name	DtF (SD)	Ratio
Taibyo-Sobutori	52.4 (9.4)	5/5
Sakurakaze	66.6 (18.1)	5/5
Top Runner	71.0 (–)	1/5
Daishi	N (–)	0/5
Shigatsu-Wase	53.0 (16.7)	3/5
New Comet	35.0 (2.1)	5/5
Ruby Comet	37.4 (2.6)	5/5
TDA-749	55.8 (18.3)	5/5
TDA-794	41.6 (4.8)	5/5
TDA-777	69.0 (15.5)	4/5
Natsu-no-Kami	35.2 (1.8)	5/5
Haru-no-Kami	39.2 (1.1)	5/5
Natsu-Tsukasa	63.4 (15.8)	5/5
NIKURA	51.3 (9.6)	4/4
Takamiya	83.0 (–)	1/5
Yume-Homare	72.0 (8.7)	4/5
Fuku-Homare	N (–)	0/5
Taka-Homare	49.3 (7.4)	4/5
Aki-Komachi	54.8 (5.8)	5/5
Sakura-no-Toride	46.8 (7.6)	5/5
Haru-Utage	49.8 (5.9)	4/5
Super-Maxim	36.0 (5.6)	5/5
Akayukichan	29.4 (2.2)	5/5
Osaka-Shijunichi	38.8 (5.7)	4/5
Kameido	31.4 (5.9)	5/5
Kuroba-Sobutori-Mino-Wase	35.8 (3.4)	5/5
Horyo	34.0 (2.6)	5/5
Moriguchi	32.8 (3.5)	4/4
Miyashige-Sobuto	33.8 (2.8)	5/5
Kotabe	30.7 (2.3)	3/3
Akasuji	31.8 (4.9)	5/5
Togakushi	39.2 (18.6)	5/5
Okute-Sakurajima	34.8 (7.9)	5/5
Harufuku	44.0 (-)	2/2
Okute-Chunaga-Ninengo	52.0 (21.7)	4/5
Choko-Aonaga	40.2 (3.9)	5/5
Choan-Aomaru-Koshin	42.4 (2.8)	5/5
Shunjyu-Altari	61.8 (16.8)	4/5
Spanish Black Round	40.6 (6.2)	5/5
Okura	28.2 (2.7)	5/5
Aokubi-Miyashige	34.0 (3.0)	5/5
Wakayama	28.2 (1.6)	5/5
Hanashirazu-Tokinashi	51.0 (8.9)	4/5

Table presents the date of first flowering within 83 days after sowing. “DtF: Days to Flower” indicates the average number of days to flowering based on the flowering individuals among the individuals used. “N” indicates that flowering did not occur. SD was calculated only for accessions in which three or more plants flowered; when two or fewer plants flowered, SD was not calculated and is indicated by “–” in the table. “Rate” represents number of flowering plants out of the individuals used.

**Table 3.** Vernalization-independent flowering of *Brassica rapa* and *Raphanus sativus* occurs under various photoperiod conditions

*B. rapa*	22 hr	11/1 hr	20 hr	18 hr	17 hr	16 hr
Musou	5/5	10/10	9/9	4/9	0/10	0/5
Chihiri-70	5/5	10/10	9/9	9/10	0/10	0/5
Kigokoro-85	5/5	10/10	9/9	0/10	0/10	0/5
Haruwarai	5/5	10/10	0/10	0/10	0/10	0/5
Eikun	5/5	10/10	10/10	7/10	0/10	0/5
Osaka-shirona	5/5	10/10	9/10	9/9	8/10	5/5
*R. sativus*	22 hr	11/1 hr	20 hr	18 hr	17 hr	16 hr
Haru-no-Kami	5/5	10/10	9/10	1/10	0/10	0/5
Hanashirazu-Tokinashi	5/5	1/10	6/10	0/10	0/10	0/5
Taibyo-Sobutori	5/5	8/10	4/10	2/10	1/8	0/5
Aki-Komachi	5/5	10/10	9/9	8/10	4/10	0/5
Natsu-Tsukasa	5/5	8/10	6/10	4/10	0/10	0/5
Sakurakaze	5/5	9/9	10/10	4/10	4/10	0/5

Table presents the percentage of individual plants that flowered without vernalization within 107 days after sowing is shown for *B. rapa* and *R. sativus* under each photoperiod condition. The number of flowering individuals is shown out of the individuals used. “11/1 hr” indicates a cycle of 11 hours of light followed by 1 hour of darkness.

## References

[B1] Bastow, R., J.S. Mylne, C. Lister, Z. Lippman, R.A. Martienssen and C. Dean (2004) Vernalization requires epigenetic silencing of *FLC* by histone methylation. Nature 427: 164–167. 10.1038/nature02269 14712277

[B2] Burn, J.E., D.J. Bagnall, J.D. Metzger, E.S. Dennis and W.J. Peacock (1993) DNA methylation, vernalization, and the initiation of flowering. Proc Natl Acad Sci USA 90: 287–291. 10.1073/pnas.90.1.287 PMC45645 11607346

[B3] Cheon, C., Y. Watanabe and T. Saito (2004) An approach to the characterization of varietal difference in inflorescence formation due to vernalization in radish plants using “Flower formation index”. Environ Control Biol 42: 305–314 (in Japanese with English summary).

[B4] Choi, K., J. Kim, H.-J. Hwang, S. Kim, C. Park, S.Y. Kim and I. Lee (2011) The FRIGIDA complex activates transcription of *FLC*, a strong flowering repressor in *Arabidopsis*, by recruiting chromatin modification factors. Plant Cell 23: 289–303. 10.1105/tpc.110.075911 PMC3051252 21282526

[B5] Helliwell, C.A., C.C. Wood, M. Robertson, W.J. Peacock and E.S. Dennis (2006) The *Arabidopsis* FLC protein interacts directly in vivo with *SOC1* and *FT* chromatin and is part of a high-molecular-weight protein complex. Plant J 46: 183–192. 10.1111/j.1365-313X.2006.02686.x 16623882

[B6] Hou, J.W., X. Cai, Y. Xu, J. Kang, X. Wang and J. Wu (2025) Identification of *BoFLC2* as a major gene controlling flowering time in cabbage (*Brassica oleracea* var. *capitata*). Sci Hortic 351: 114407.

[B7] Kitamoto, N., S. Yui, K. Nishikawa, Y. Takahata and S. Yokoi (2014) A naturally occurring long insertion in the first intron in the *Brassica rapa* *FLC2* gene causes delayed bolting. Euphytica 196: 213–223.

[B8] Lang, A. (1957) The effect of gibberellin upon flower formation. Proc Natl Acad Sci USA 43: 709–717. 10.1073/pnas.43.8.709 PMC528525 16590073

[B9] Leijten, W., R. Koes, I. Roobeek and G. Frugis (2018) Translating flowering time from *Arabidopsis thaliana* to Brassicaceae and Asteraceae crop species. Plants 7: 111. 10.3390/plants7040111 PMC6313873 30558374

[B10] Motoki, K., Y. Kinoshita and M. Hosokawa (2019) Non-vernalization flowering and seed set of cabbage induced by grafting onto radish rootstocks. Front Plant Sci 9: 1967. 10.3389/fpls.2018.01967 PMC6335391 30687362

[B11] Samach, A., H. Onouchi, S.E. Gold, G.S. Ditta, Z. Schwarz-Sommer, M.F. Yanofsky and G. Coupland (2000) Distinct roles of CONSTANS target genes in reproductive development of *Arabidopsis*. Science 288: 1613–1616. 10.1126/science.288.5471.1613 10834834

[B12] Searle, I., Y. He, F. Turck, C. Vincent, F. Fornara, S. Kröber, R.A. Amasino and G. Coupland (2006) The transcription factor FLC confers a flowering response to vernalization by repressing meristem competence and systemic signaling in *Arabidopsis*. Genes Dev 20: 898–912. 10.1101/gad.373506 PMC1472290 16600915

[B13] Shindo, C., M.J. Aranzana, C. Lister, C. Baxter, C. Nicholls, M. Nordborg and C. Dean (2005) Role of FRIGIDA and *FLOWERING LOCUS C* in determining variation in flowering time of Arabidopsis. Plant Physiol 138: 1163–1173. 10.1104/pp.105.061309 PMC1150429 15908596

[B14] Song, Y.H., A. Kubota, M.S. Kwon, M.F. Covington, N. Lee, E.R. Taagen, D.L. Cintrón, D.Y. Hwang, R. Akiyama, S.K. Hodge et al. (2018) Molecular basis of flowering under natural long-day conditions in *Arabidopsis*. Nat Plants 4: 824–835. 10.1038/s41477-018-0253-3 PMC6195122 30250277

[B15] Valverde, F., A. Mouradov, W. Soppe, D. Ravenscroft, A. Samach and G. Coupland (2004) Photoreceptor regulation of CONSTANS protein in photoperiodic flowering. Science 303: 1003–1006. 10.1126/science.1091761 14963328

[B16] Wang, Q., Y. Zhang and L. Zhang (2018) A naturally occurring insertion in the *RsFLC2* gene associated with late-bolting trait in radish (*Raphanus sativus* L.). Mol Breed 38: 137.

[B17] Watson, A., S. Ghosh, M.J. Williams, W.S. Cuddy, J. Simmonds, M.D. Rey, M.A. Md Hatta, A. Hinchliffe, A. Steed, D. Reynolds et al. (2018) Speed breeding is a powerful tool to accelerate crop research and breeding. Nat Plants 4: 23–29. 10.1038/s41477-017-0083-8 29292376

[B18] Yui, S., K. Hida and Y. Kuginuki (2003) Breeding of ‘Leafy green parental line No. 2’, long-day requiring late bolting material. Breed Res 5: 169–173 (in Japanese).

